# P-2205. The impact of pharmacist-driven transitions of care protocol in the treatment of hepatitis C infection

**DOI:** 10.1093/ofid/ofae631.2359

**Published:** 2025-01-29

**Authors:** Sophea Chan, Christo Cimino, Austin Ing, Benjamin Ereshefsky, Laura Bobbitt, Ryan Moss, Anahit Muscarella

**Affiliations:** Vanderbilt University Medical Center, Nashville , Tennessee; Vanderbilt University Medical Center, Nashville, Tennessee; Vanderbilt University Medical Center, Nashville, Tennessee; Vanderbilt University Medical Center, Nashville, Tennessee; Vanderbilt University Medical Center, Nashville, Tennessee; Vanderbilt University Medical Center, Nashville, Tennessee; Vanderbilt University Medical Center, Nashville, Tennessee

## Abstract

**Background:**

Treatment for hepatitis C virus (HCV) can be delayed due to extensive documentation needed for insurance approval. Pharmacists can play critical roles in facilitating treatment approval, but the evidence regarding pharmacist-driven transitions of care protocols to facilitate HCV treatment approval and therapy initiation is limited. Therefore, we evaluated the feasibility and effectiveness of a pharmacist-driven transitions of care protocol for HCV treatment.

**Methods:**

This was a non-randomized, prospective, single-center study at a large academic medical center. Adult patients admitted to the Infectious Diseases (ID) primary team were screened for HCV between 09/01/23 and 02/29/24. Exclusion criteria included decompensated cirrhosis, hepatocellular carcinoma, pregnancy/breastfeeding, current/prior HCV treatment, and transplant evaluation/receipt. Patients with HCV infection were offered treatment upon discharge at the outpatient ID clinic. Inpatient ID pharmacists performed pre-treatment workup and coordinated handoff to outpatient ID specialty pharmacists. Endpoints included treatment initiation and completion rates, time from screening to treatment initiation, and time from discharge to treatment initiation.

**Results:**

Of 180 patients screened during the study, 13 (7%) were eligible for HCV treatment. The median age was 43 years, and 69% were male. More than 90% of patients were Caucasian and had a history of substance use disorder/injection drug use. Five patients (38%) were uninsured. Of the 13 patients, two had treatment approved, and one completed therapy. The remaining patients were lost to follow-up. The median times from screening to treatment initiation and from discharge to treatment initiation were 64 and 62 days, respectively.

**Conclusion:**

This pharmacist-driven protocol resulted in inpatient screening of a large number of patients over six months and successfully identified 13 patients eligible to begin hepatitis C treatment after discharge. This is a novel approach to engaging patients in hepatitis C care. Despite facilitating transitions of care for these patients, the clinic no-show rate was unexpectedly high. Additional measures may be required to optimize treatment outcomes for this patient population.

**Disclosures:**

All Authors: No reported disclosures

